# The role of laparoscopic surgery in the surgical management of recurrent liver malignancies: A systematic review and meta-analysis

**DOI:** 10.3389/fsurg.2022.1042458

**Published:** 2023-01-06

**Authors:** Tian-Run Lv, Hai-Jie Hu, Wen-Jie Ma, Ya-Fei Hu, Yu-Shi Dai, Fu-Yu Li

**Affiliations:** Department of Biliary Surgery, West China Hospital, Sichuan University, Chengdu, China

**Keywords:** laparoscopic, hepatectomy, recurrent, liver resection, minimally invasive

## Abstract

**Objective:**

To evaluate the efficiency of laparoscopic surgery in treating recurrent liver tumors vs. conventional open surgery.

**Methods:**

Database searching was conducted in PubMed, the Cochrane Library and EMBASE. Rev Man 5.3 software and Stata 13.0 software were applied in statistical analyses.

**Results:**

A total of fourteen studies were finally included with 1,284 patients receiving LRH and 2,254 with ORH. LRH was associated with less intraoperative hemorrhage, a higher R0 resection rate, a lower incidence of Pringle Maneuver, a lower incidence of postoperative morbidities, a better overall survival and an enhanced postoperative recovery vs. ORH. Patients receiving LRH shared similar operative time, tumor number and disease-free survival as those with ORH. However, tumor size was relatively larger in patients receiving ORH and major hepatectomy, anatomic hepatectomy were rarely performed in patients with LRH. Additional analyses between LRH and laparoscopic primary hepatectomy revealed less intraoperative blood loss in patients with LRH.

**Conclusion:**

LRH is safe and feasible with more favorable peri-operative outcomes and faster postoperative recovery. However, it is only applicable for some highly-selected cases not requiring complex surgical procedures. Future larger well-designed studies are expected for further validation.

## Introduction

Hepatectomy with a preserved liver function has been widely applied in the curative treatment of primary liver malignancies, such as hepatocellular carcinoma (HCC), intrahepatic cholangiocarcinoma (IHCC) and colorectal liver metastases (CRLM) (1–3). However, even after radical resection, the incidence of recurrent liver disease remains high with a high recurrence rate reaching 80% for patients with HCC (2). Therefore, regarding recurrent liver malignancies, effective therapeutic modalities are demanded to prolong the overall survival as much as possible. Currently, various modalities have been developed, including hepatectomy, trans arterial chemoembolization, ablation as well as systematic adjuvant therapies. Repeat hepatectomy with a favorable preserved liver function has been demonstrated to be especially effective with a promising prognosis in patients with recurrent liver disease (4–6).

Currently, minimally invasive surgery (MIS), especially laparoscopic surgery (LS), has been widely applied in the surgical management of various benign or malignant diseases. Laparoscopic hepatectomy (LH) has acquired unexpected superior peri-operative outcomes vs. conventional open surgery in patients with minor or solitary liver tumors (7–9). Nevertheless, when it comes to recurrent liver disease, laparoscopic repeat hepatectomy (LRH) can be technically challenging. Owing to the adhesions after the previous surgery, anatomic resections can be difficult and would take a great risk of unintended vascular or biliary injuries. Pringle maneuver, an effective method in controlling intraoperative blood loss, would be also hard to apply due to tense adhesions around the hepatoduodenal ligament (HDL), which would cause a high conversion rate. However, over the last decade, numerous studies (10–16) have focused on LRH and acquired promising results vs. conventional open repeat hepatectomy (ORH), including fewer postoperative complications, less intraoperative blood loss and shorter postoperative hospital stay. However, the limited sample size and the incomplete evaluation have greatly undermined the validity of their results and conclusions (10, 13–16). Recently, a propensity scoring matching study and meta-analysis (11) focusing on this debating issue concluded that LRH acquired better surgical outcomes and an enhanced postoperative recovery. However, there were fatal defects in their analysis that the data in their study as well as another most-recently published study (17) was not incorporated. Specific surgical procedures related to the applicability of LRH, such as anatomic resection and major hepatectomy, and long-term prognosis were not furtherly analyzed.

Hence, a more powerful evaluation on the safety and feasibility of LRH vs. ORH is required and our meta-analysis was performed to explore this elusive issue in terms of intra and postoperative outcomes and long-term prognosis.

## Materials and methods

### Search strategy

The PRISMA Statement for Reporting Systematic Reviews and Meta-Analyses of Studies That Evaluate Health Care Interventions: Explanation and Elaboration (18) is the basic items for our study to follow. PubMed, the Cochrane library and EMBASE were searched till August 1^st^ 2022. The following keywords were used for literature searching: (((repeat hepatectomy) OR (repeat liver resection)) OR (recurrent)) AND ((minimally invasive) OR (laparoscpic)).

### Inclusion criteria and exclusion criteria

(1) Published English literatures
(2) Any comparative study between LRH and ORH
(3) Studies reported intraoperative or peri-operative outcomes or long-term survival.
(4) Studies which have provided adequate date for further analysis.
(5) Studies shared a completely same database or patients source.
(6) Abstracts, letters, meeting conference or reviews.

### Quality assessment and statistical analyses

The specific modalities within our manuscript regarding quality evaluation of identified studies and statistical analyses are similar to our previous series (19). In order to reduce similarity index, no more illustrations will be provided (Table 1).

**Table 1:** Baseline characteristics of all studies included.

Author	Study period	Study design	Region	No. Patients		Pathology	Conversion *n* (%)	Follow up (months)	Quality Score (NOS)
				LRH	ORH				
Kanazawa A et al	2006–2011	Retrospective	Osaka City General Hospital, Osaka, Japan	20	20	HCC	2 (10%)	NA	6
Chan ACY et al	2004–2013	Retrospective	Queen Mary Hospital, Hong Kong	11	22	HCC	0 (0%)	3 months in the first 2 years and 6 months thereafter	7
Zhang J et al	2014.6–2014.9	Prospective	Sun Yat-sen University Cancer Center, China	31	33	HCC	0 (0%)	Median 17, range (12–18)	7
Hallet J et al	2006–2013	Retrospective	AFC CRLM database, French	27	349	CRLM	1 (3.1%)	Median 20.7, (IQR: 3.3–60.2)	8
Liu K et al	2008–2015	Retrospective	Sun Yat-Sen Memorial Hospital, China	30	30	HCC	4 (13.3%)	Median 35, range (2–80)	8
Noda T et al	2005–2016	Retrospective	Osaka Police Hospital, Osaka, Japan	20	48	HCC	1 (5%)	NA	7
Ome Y et al	2010–2017	Retrospective	Kurashiki Central Hospital, Japan	33	37	HCC + IHCC + HCC-CC + CRLM + others	0 (0%)	NA	7
Goh BPK et al	2015–2017	Retrospective	Singapore General Hospital, Singapore	20	79	HCC	3 (15%)	NA	7
Poel MJ et al	2000–2016	Retrospective	Nine highly experienced hepato-pancreato-biliary centres from seven European countries	271	154	CRLM	30 (11.1%)	NA	7
Inoue Y et al	2010–2018	Retrospective	Osaka Medical College Hospital, Japan	45	97	HCC + IHCC + others	6 (13.3%)	NA	7
Morise Z et al	2007–2017	Retrospective	Forty-two liver surgery centers around the world	648	934	HCC	NA	NA	7
Onoe T et al	2007–2018	Retrospective	Kure Medical Center and Chugoku Cancer Center, Japan	30	42	HCC	2 (6.75%)	NA	7
Chen JF et al	2017.01–2018.12	Retrospective	Zhong shan Hospital, Shanghai, China	68	358	HCC	6 (8.82%)	NA	8
Gon H et al	2008–2019	Retrospective	Graduate School of Medicine, Kobe University, Japan	30	51	HCC	2 (6.67%)	Every 3 months	8

LRH, laparoscopic repeat hepatectomy; ORH, open repeat hepatectomy; CRLM, colorectal liver metastases; AFC, French Surgical Association; No. patients: the number of patients; HCC, hepatocellular carcinoma; IHCC, intrahepatic cholangiocarcinoma; HCC-CC, combined hepatocellular carcinoma and cholangiocarcinoma; NA, not available; IQR, interquartile range; NOS, Newcastle– Ottawa Quality Assessment Scale.

## Results

### Study identification and selection

**Figure 1:**
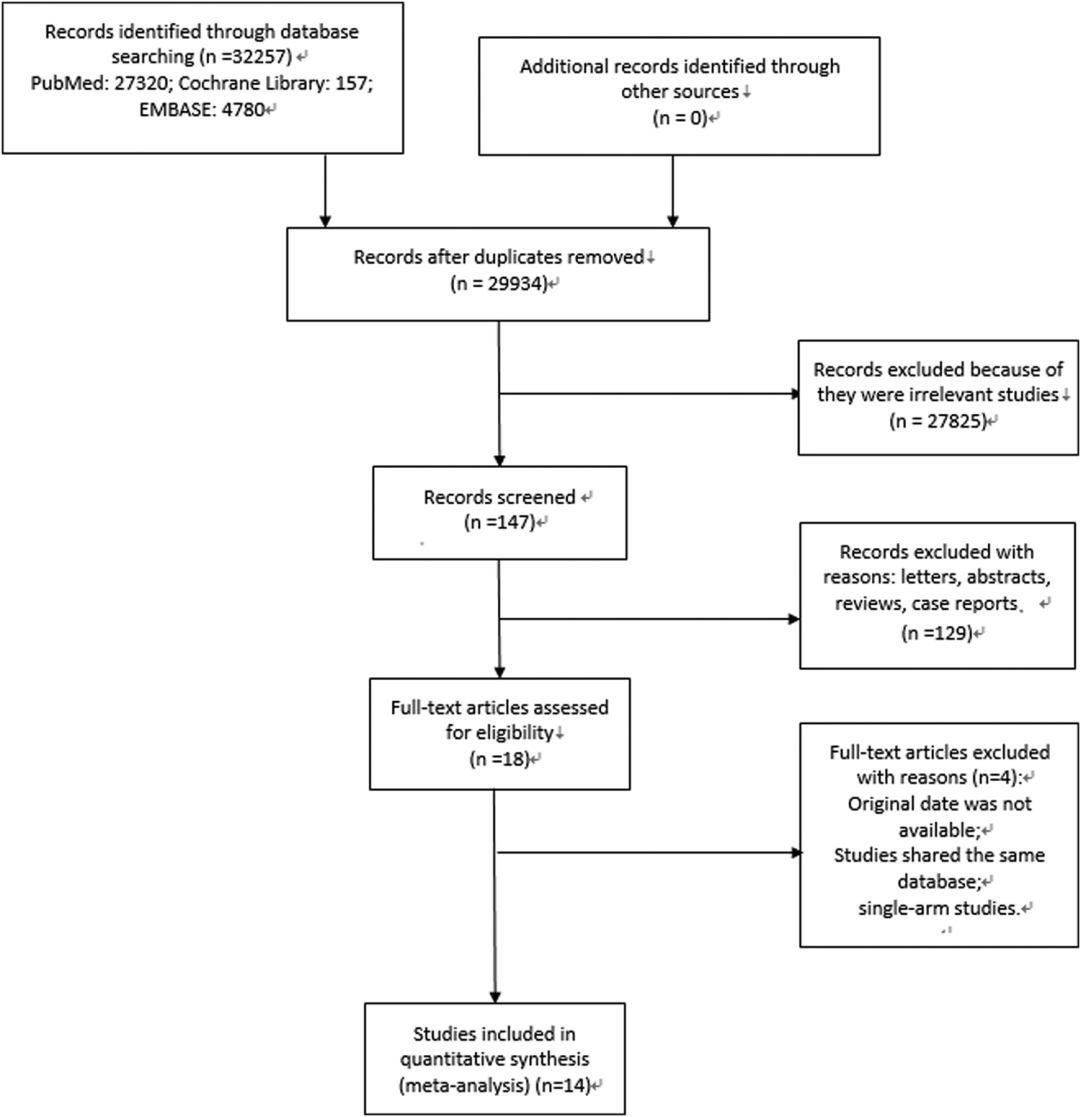
Specific process of literature researching and selection.

At the beginning, 2,561 relevant articles were retrieved and after the inclusion and exclusion process, fourteen studies were finally included. The specific process is depicted in Figure 1.

### Study characteristics

A total of fourteen studies (10–12, 14, 17, 20–28) were finally identified with 1,284 patients receiving LRH and 2,254 patients receiving ORH. All studies except for the study by Zhang J et al. (28) were retrospective cohort studies. Pathologies of liver tumors included HCC, IHCC, HCC-CC and CRLM (Table 1). The majority of studies included only reported pero-operative details, including blood loss, intraoperative time, tumor size and postoperative hospital stay. Only six studies (10, 12, 14, 20, 23, 28) reported the postoperative long-term survival. A total of twelve measured parameters were finally identified, including operative time, intraoperative blood loss, tumor size (continuous), multiple tumors, major hepatectomy (≥3 segments), anatomic hepatectomy, pringle maneuver, R0 resection rate, postoperative morbidities, hospital stay, overall survival (OS) and disease-free survival (DFS) (Table 2). Moreover, the study by Ome Y et al. (25) and the study by Goh BKP et al. (12) also reported similarities and differences between the laparoscopic primary hepatectomy (LPH) and LRH. Consequently, we also compared LRH and LPH accordingly (Supplementary Table S1). Considering the inconsistencies of surgical indication between laparoscopic and open surgery, we have also collected the surgical indication of LRH in each literature (Supplementary Table S2).

**Table 2:** Pooled results of all available studies in measured outcomes.

Outcomes	No. studies	No. patients		Model (Fixed/random)	OR/HR/WMD	95% CI	*P* (overall test)	P^C^ (overall test)	Heterogeneity		Begg's test		Egger's test
		LRH	ORH						*I*^2^ (%)	*P*	Pr>|z|*	Pr >|z|**	*P*>|t|*
Operative time	14	1258	1926	Random	WMD = −8.17	−34.83–18.50	*P* = 0.55	*P*^C ^= 0.55	94%	<0.00001	0.870	0.913	0.691
Intraoperative blood loss	13	1231	1577	Random	WMD = −281.21	−361.53–200.90	*P* < 0.00001	*P*^C ^< 0.00001	93%	<0.00001	0.088	0.100	0.335
Tumor size (continuous)	11	1201	1786	Random	WMD = −0.55	−0.77–−0.33	*P* < 0.00001	*P*^C ^< 0.00001	76%	<0.0001	0.484	0.533	0.578
Tumor number (≥2, dichotomic variables)	6	382	325	Fixed	OR = 0.71	0.50–1.00	*P* = 0.05	*P*^C ^= 0.05	49%	=0.08	0.851	1	0.986
Major hepatectomy (≥3 segments)	6	1026	1597	Random	OR = 0.64	0.16–2.54	*P* = 0.52	*P*^C ^= 0.008	92%	<0.00001	0.573	0.707	0.478
Anatomic hepatectomy	5	384	332	Fixed	OR = 0.43	0.31–0.61	*P* < 0.00001	*P*^C ^< 0.00001	8%	*P* = 0.36	0.142	0.221	0.638
Pringle maneuver	6	425	717	Random	OR = 0.22	0.11–0.43	*P* < 0.0001	*P*^C ^< 0.00001	54%	*P* = 0.05	0.851	1	0.642
R0 resection	3	311	213	Fixed	OR = 2.78	1.62–4.74	*P* = 0.0002	*P*^C ^= 0.0002	0%	*P* = 0.72	0.602	1	0.592
Postoperative morbidities	11	1185	1520	Fixed	OR = 0.54	0.43–0.69	*P* < 0.00001	*P*^C ^< 0.00001	48%	*P* = 0.04	0.586	0.640	0.083
Postoperative hospital stay	13	1231	1577	Random	OR = −3.10	−3.84–2.37	*P* < 0.00001	*P*^C ^< 0.00001	88%	<0.00001	0.067	0.077	0.085
Overall survival	3	279	289	Fixed	HR = 0.62	0.44–0.86	*P* = 0.004	*P*^C ^= 0.004	0%	=0.98	0.602	1	0.885
Disease-free survival	6	352	472	Fixed	HR = 1.02	0.82–1.26	*P* = 0.88	*P*^C ^= 0.88	0%	=0.48	0.573	0.707	0.535

No.: the number of; LRH, laparoscopic repeat hepatectomy; ORH, open repeat hepatectomy; OR, odds ratio; HR, hazard ratio; WMD, weighted mean difference; CI, confidence interval; *P*^C^, corrected *P* value.

* *P*-value.

** *P*-value (continuity corrected); *P*^C^: corrected *P* value after the sensitivity analysis.

**Figure 2:**
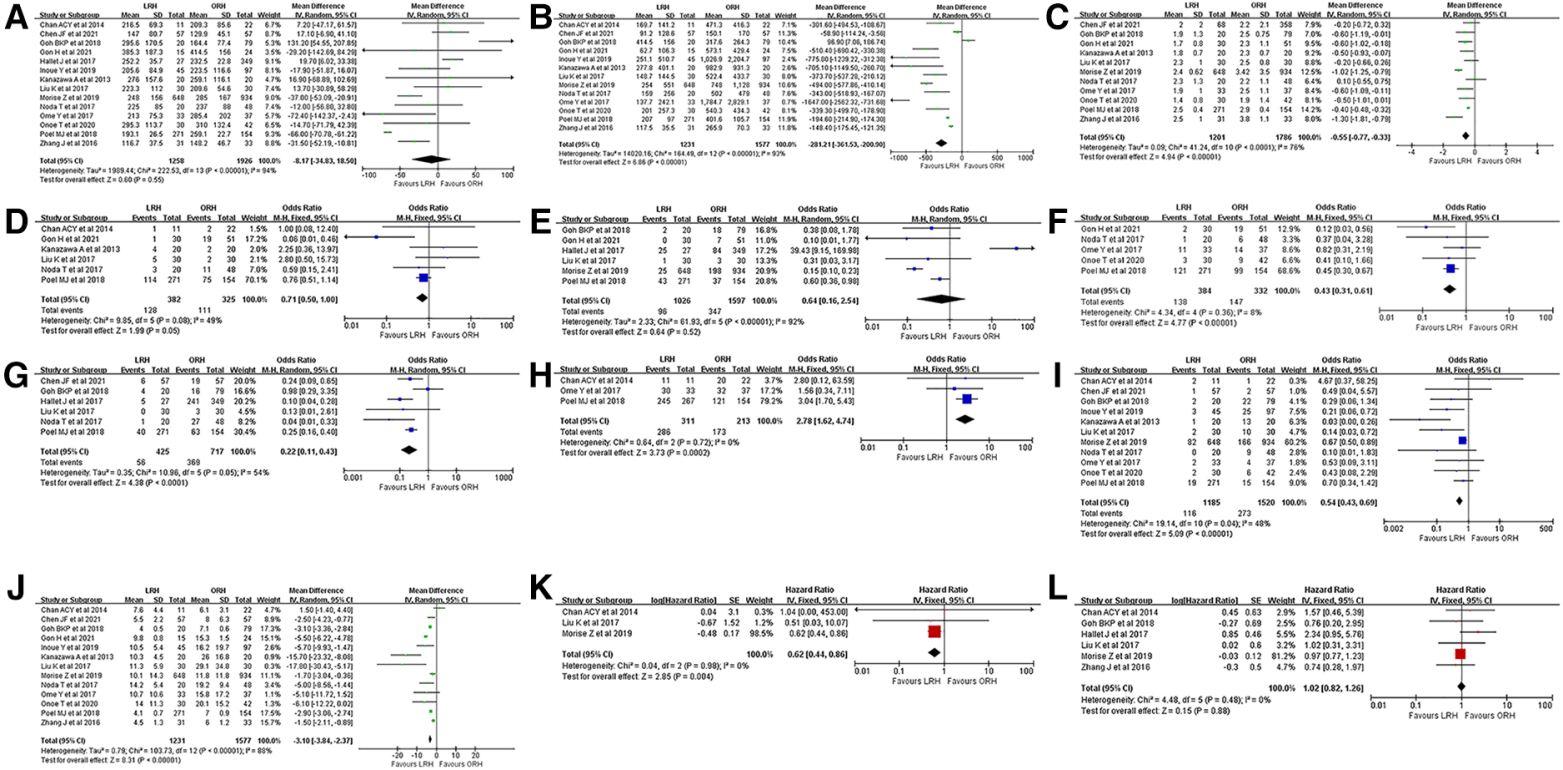
Forest plots presenting the intraoperative, postoperative and survival outcomes. (**A**), operative time. (**B**), intraoperative blood loss. (**C**), tumor size (continuous). (**D**), multiple tumors. (**E**), major hepatectomy (≥3 segments). (**F**), anatomic hepatectomy. (**G**), pringle maneuver. (**H**), R0 resection rate. (**I**), postoperative morbidities. (**J**), hospital stay. (**K**), overall survival (OS). (**L**), disease-free survival (DFS).

**Operative time:** All studies included reported the operative time of LRH and ORH respectively and pooled data from fourteen studies revealed no significant difference between LRH and ORH (WMD = −8.17; 95% CI −34.83 to 18.50; *P* = 0.55) (*χ*2 = 222.53, *P* < 0.00001, *I*^2^ = 94%) (Figure 2A).

**Intraoperative blood loss:** Thirteen studies reported the intraoperative blood loss and pooled date revealed significantly less intraoperative loss in the LRH group (WMD = −281.21; 95% CI −361.53 to −200.90; *P* < 0.00001) (*χ*2 = 164.49, *P* < 0.00001, *I*^2^ = 93%) (Figure 2B).

**Tumor size (continuous):** Eleven studies reported the resected tumor size and pooled data revealed an extremely larger tumor size in the ORH group (WMD = −0.55; 95% CI −0.77 to −0.33; *P* < 0.00001) (*χ*2 = 41.24, *P* < 0.0001, *I*^2^ = 76%) (Figure 2C).

**Tumor number (≥2):** Six studies reported the number of patients with multifocal lesions and pooled data revealed no significant difference on the percentage of patients with multifocal tumors in LRH and ORH groups (32.5% vs. 34.2%, OR=0.71, 95% CI 0.50 to 1.00; *P* = 0.05) (*χ*2 = 9.85, *P* = 0.08, *I*^2^ = 49%) (Figure 2D).

**Major hepatectomy (≥3 segments):** Six studies were incorporated and pooled data revealed a higher rate with a borderline *P* value in the ORH group (9.4% vs. 21.7%, OR=0.64, 95% CI 0.16 to 2.54; *P* = 0.52) (*χ*2 = 61.93, *P* < 0.00001, *I*^2^ = 92%) (Figure 2E). However, after the sensitivity analysis, a statistical difference was acquired when the study by Hallet J et al. (20) was excluded (7.1% vs. 21.1%, OR=0.28, 95% CI 0.11 to 0.72; *P* = 0.008).

**Anatomic hepatectomy:** Five studies were incorporated and the pooled date revealed a significantly higher incidence in the ORH group (36.0% vs. 44.3%, OR=0.43, 95% CI 0.31 to 0.61; *P* < 0.00001) (*χ*2 = 4.34, *P* = 0.36, *I*^2^ = 8%) (Figure 2F).

**Pringle Maneuver:** Six studies reported the number of patients receiving Pringle Maneuver and the pooled data revealed a significantly higher rate in the ORH group (13.2% vs. 51.5%, OR=0.22, 95% CI 0.11 to 0.43; *P* < 0.0001) (*χ*2 = 10.96, *P* = 0.05, *I*^2^ = 54%) (Figure 2G). Significant heterogeneity was detected and after the heterogeneity analysis, a corrected *P* value with a significantly lower heterogeneity was acquired (*P* < 0.00001, *χ*2 = 5.16, *P* = 0.27, *I*^2^ = 23%) when the study by Goh BKP et al. (12) was excluded.

**R0 resection:** Three studies were incorporated and the pooled result revealed a significantly higher incidence in the LRH group (92.0% vs. 81.2%, OR=2.78, 95% CI 1.62 to 4.74; *P* = 0.0002) (*χ*2 = 0.64, *P* = 0.72, *I*^2^ = 0%) (Figure 2H).

**Postoperative morbidities:** Eleven studies were incorporated and the pooled data revealed a significantly lower rate in the LRH group (9.8% vs. 18.0%, OR=0.54, 95% CI 0.43 to 0.69; *P* < 0.00001) (*χ*2 = 19.14, *P* = 0.04, *I*^2^ = 48%) (Figure 2I).

**Postoperative hospital stay:** Thirteen studies were incorporated and the pooled result revealed that patients receiving LRH recovered much faster than those with ORH (WMD = −3.10; 95% CI −3.84 to −2.37; *P* < 0.00001) (*χ*2 = 103.73, *P* < 0.00001, *I*^2^ = 88%) (Figure 2J).

**OS:** Three studies were incorporated and the pooled result revealed a significantly better OS in patients receiving LRH (HR = 0.62, 95% CI 0.44 to 0.86, *P* = 0.004) (Figure 2K).

**DFS:** Six studies were incorporated and the pooled result revealed a similar DFS between two groups (HR = 1.02, 95% CI 0.82 to 1.26, *P* = 0.88) (Figure 2L).

### Additional analyses between LPH and LRH

As was presented in Supplementary Table S1 and Supplementary Figure S1, only two studies (12, 25) analyzed the consistencies and inconsistencies between LPH and LRH. Only five measured outcomes were identified and pooled results revealed that LPH was similar to LRH in terms of conversion rate, operative time, postoperative complications and postoperative hospital stay. However, the intraoperative blood loss was significantly lower in LRH group (*P* = 0.03).

### Publication bias, heterogeneity analysis and sensitivity analysis

As was summarized in Table 2, after a systematic statistical analysis, all the *P* values in the Begg's test or corrected *P* values in the Egger's test were greater than 0.05, indicating the absence of remarkable bias. The results of heterogeneity analysis and sensitivity analysis were presented in the **Results** section.

## Discussion

Current study is an updated systematic review and meta-analysis comparing the efficiency of laparoscopic surgery in the surgical management of recurrent liver tumors vs. conventional open surgery. Although the most-recently published Propensity Score–Matched Study and Meta-Analysis by Chen JF et al. (11) has collected relevant information and concluded that LRH was safe and feasible for recurrent liver tumors, their study is less convincing owing to the following reasons. First, their analysis only included intraoperative details, such as blood loss or operative time, rather, surgical procedures and postoperative survival were neglected. Second, their own study was not regarded as one of the included studies and another recently-published study by Gon H et al. (17) was not incorporated as well. Third, a total of twelve measured parameters were finally identified in our analysis while only six outcomes were observed in their results. A more comprehensive re-evaluation has been carried out and our major findings were as follows:

(1) Laparoscopic surgery seems to be more safe and feasible for recurrent liver tumors with less intraoperative hemorrhage, a lower incidence of Pringle Maneuver, a lower incidence of postoperative morbidities, faster postoperative recovery, and a better OS.
(2) The surgical indication of LRH differed a lot from ORH that LRH was more frequently applied in patients with smaller tumor size. Major hepatectomy and anatomic hepatectomy were rarely performed *via* laparoscopic approach while they were common in open surgery.
(3) LRH was superior to LPH with less intraoperative hemorrhage.

Owing to the rapid evolvement of minimally invasive technique, laparoscopic surgery has been widely applied in the surgical management of various cancers, such as gastric cancer and colorectal cancer (29–31). The establish of pneumo-peritoneum and a magnified view *via* laparoscopic approach greatly induced the intraoperative hemorrhage, allowing surgeons to operate subtly without unnecessary injuries to adjacent organs and structures. Moreover, as was observed in our analysis as well as in many other published literatures (10–16), patients receiving LRH often exhibited an enhanced recovery. Pringle maneuver is a potentially possible reason causing the difference of the postoperative recovery. The application of Pringle maneuver was more frequently detected in the ORH group (*P* < 0.05), which may result in ischemia–reperfusion injury and post-surgical hepatic dysfunction (32). The postoperative inflammation process would also have an impact on the recovery time for the inflammation-based markers have been demonstrated with great elevation in patients receiving ORH in the study by Chen JF et al. (11). They even proved that SII ≤ 431.7 on POD3 was associated with shorter hospital stay, suggesting its value in predicating enhanced recovery.

Apart from the promising aspects of LRH in intraoperative blood loss, the application of Pringle maneuver and the enhanced postoperative recovery, a significantly higher R0 resection rate was also observed in our results (*P* = 0.0002). A curative-intent surgery with a negative margin has always been regarded as an effective method in evaluating the surgical efficiency. In other words, LRH achieved a more favorable tumor clearance than ORH. However, it's still not reasonable to draw a conclusion that LRH is superior to ORH due to numerous unavoidable factors. As was observed in our analysis and many other studies, the candidates for LRH are usually highly-selected and well-prepared. For example, major hepatectomy and anatomic liver resection were rarely performed in patients receiving LRH but they were common in patients with ORH (*P* < 0.05). Such phenomenon has also been validated in many other published studies (12, 17, 20, 23), suggesting that LRH may be safe and feasible in patients not requiring complex surgical procedures. Recently, Chen JF et al. (11) reported the successful application of LRH in posterosuperior segments or tumor size larger than 5 cm. However, their small sample size was unable to reverse the trend, not to mention to draw a powerful conclusion. We have also collected the inclusion criteria of patients receiving LRH among the studies included in our analysis and surprisingly found that the majority of candidates for LRH were characterized as a favorable preserved liver function (Child A or B), without major vessels or bile duct invasion and with a solitary mass (Supplementary Table S2). The observations above reflected the fact that LRH is still not universally applicable.

The initial exploration regarding the application of laparoscopic surgery in recurrent liver malignancies can date back to 2013. Kanazawa A et al. (22) firstly compared 20 patients receiving LRH vs. 20 with ORH. The significant selection bias that the incidence of intractable ascites was significantly higher in patients receiving ORH (*P* = 0.0436) greatly weakened the validity of their conclusion. Four years later, Liu K et al. (23) conducted a well-controlled study among 60 recurrent HCCs (LRH: ORH = 1:1) and revealed that laparoscopic approach was superior to open surgery in intraoperative blood loss, postoperative complications and postoperative hospital stay. However, another study (Hallet J et al.) (20) regarding CRLMs showed no significant difference in surgery-related outcomes except for the postoperative morbidities between laparoscopic and open approaches. This reverse trend can be accounted to the difference in tumor types, surgical techniques and the unavoidable selection bias owing to the retrospective nature. Both studies neglected the impact caused by tumor locations and the initial surgical approaches. Open surgery tended to cause more severe adhesions than laparoscopic surgery and would make the following laparoscopic surgery more difficult to perform (33). Fortunately, the bias mentioned above were perfectly resolved in the propensity scores matching analysis among 114 patients (LRH: ORH = 1:1) by Chen JF et al. (11). Moreover, tumor size, tumor number, preoperative liver functions (Child class) and laboratory examinations (*P* > 0.05) were also well-controlled. However, the inherent bias regarding surgical procedures, especially major hepatectomy and anatomic resection, was still unsettled. Hence, their conclusions were still less convincing and our study creatively took these factors into consideration and draw a balanced conclusion, that is, LRH does have its superiorities to ORH but is only applicable for some highly-selected patients, which was mainly due to the unbalanced proportion of patients receiving major and anatomic liver resections in the LRH and ORH groups (*P* < 0.05). Regarding the technical difficulties of LRH, the Southampton guidelines have indicated that LRH should be performed in experienced centers rather than in their preliminary stage (34). The location of trocar hole should be adjusted according to the newest liver anatomy and the adhesions formed after the previous surgery. Unnecessary adhesiolysis should also be avoided for favoring the future abdominal surgeries (35, 36). As for postoperative survival, our results revealed that patients receiving LRH had a significantly better OS than patients receiving ORH (*P* < 0.05). Further analyzing its potential reasons, we subjectively accounted it for the selection bias existed in the majority of our included studies. The candidates for LRH were often solitary, small and without major vascular invasion or biliary invasion (Supplementary Table S2). Moreover, considering the natural advantages of laparoscopic technique, including the less exposure of abdominal organs, smaller incisions, meticulous manipulations without unnecessary damage to adjacent liver parenchyma and a magnified view facilitating tumor clearance, a better OS should be ideally acquired in LRH group (14, 37, 38).

There are several limitations within our manuscript. First, owing to the nature of retrospective studies, the selection bias as well as the inconsistency on the surgical indication of LRH made our study less statistically powerful. Second, the small sample size would also make our results and conclusions less powerful. Third, the rough estimate of HR *via* Tierney's method may lead to moderate bias. Fourth, the impact caused by the approaches (laparoscopic or open) should be furtherly analyzed. However, the absence of original data might hinder us from further exploration to some extent.

## Conclusion

Our study revealed the efficiency of laparoscopic approach in treating recurrent liver malignancies. Compared with conventional open approach, LRH showed its superiority in operative time, intraoperative blood loss, R0 resection rate, Pringle Maneuver, postoperative complications and hospital stay. However, LRH was rarely performed in patients with recurrent liver tumors requiring more complex surgical procedures, such as major hepatectomy or anatomic liver resections. Therefore, we herein could only conclude that LRH does has its superiorities to ORH but it can only be only applicable to some highly-selected cases. To perform LRH, experienced centers are firstly preferred and more perfectly-designed studies are demanded for further validation.

## Data Availability

The original contributions presented in the study are included in the article/**Supplementary Material**, further inquiries can be directed to the corresponding author/s.
