# Supplementary material for: The role of laparoscopic surgery in the surgical management of recurrent liver malignancies: A systematic review and meta-analysis
Source: Front Surg. 2023 Jan 6;9:1042458. doi: 10.3389/fsurg.2022.1042458 (PMC9852625; doi:10.3389/fsurg.2022.1042458)
Supplement: Supplementary file 1 [file Datasheet1.docx]

**Figure S1**

**Figure S1A**


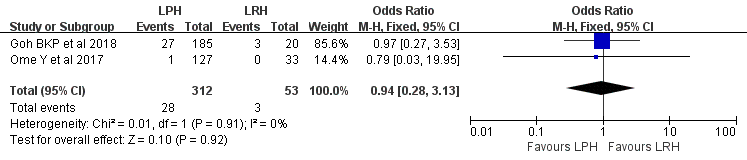


**Figure S1B**

**
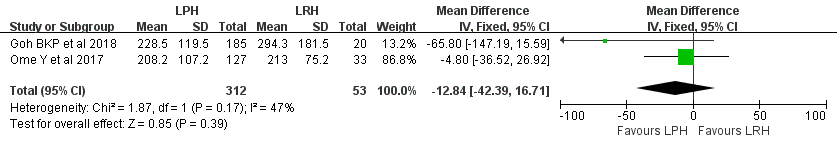
**

**Figure S1C**

**
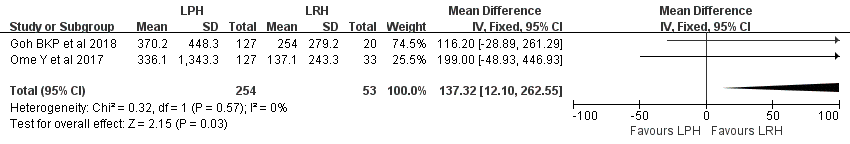
**

**Figure S1D**

**
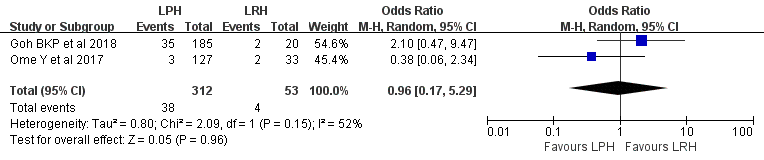
**

**Figure S1E**

**
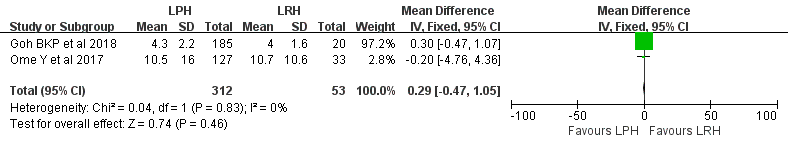
**

**Figure S1** Forest plots presenting the consistencies and inconsistencies between laparoscopic primary hepatectomy and laparoscopic repeat hepatectomy. A, conversion rate. B, operative time. C, intraoperative blood loss. D, postoperative complications. E, postoperative hospital stay.
